# Thromboses and Hemostasis Disorders Associated with COVID-19: The Possible Causal Role of Cross-Reactivity and Immunological Imprinting

**DOI:** 10.1055/s-0041-1731068

**Published:** 2021-06-26

**Authors:** Darja Kanduc

**Affiliations:** 1 Department of Biosciences, Biotechnologies and Biopharmaceutics, University of Bari, Bari, Italy

**Keywords:** COVID-19, SARS-CoV-2 spike gp, cross-reactivity, immunological imprinting, thromboses-related proteins, thromboses, vascular diseases, bleeding

## Abstract

By examining the issue of the thromboses and hemostasis disorders associated with severe acute respiratory syndrome-coronavirus-2 (SARS-CoV-2) through the lens of cross-reactivity, it was found that 60 pentapeptides are shared by SARS-CoV-2 spike glycoprotein (gp) and human proteins that— when altered, mutated, deficient or, however, improperly functioning— cause vascular diseases, thromboembolic complications, venous thrombosis, thrombocytopenia, coagulopathies, and bleeding, inter alia. The peptide commonality has a relevant immunological potential as almost all of the shared sequences are present in experimentally validated SARS-CoV-2 spike gp-derived epitopes, thus supporting the possibility of cross-reactions between the viral gp and the thromboses-related human proteins. Moreover, many of the shared peptide sequences are also present in pathogens to which individuals have previously been exposed following natural infection or vaccinal routes, and of which the immune system has stored imprint. Such an immunological memory might rapidly trigger anamnestic secondary cross-reactive responses of extreme affinity and avidity, in this way explaining the thromboembolic adverse events that can associate with SARS-CoV-2 infection or active immunization.

## Introduction

Clinical studies have shown that severe acute respiratory syndrome-coronavirus-2 (SARS-CoV-2) infection can lead to an increased incidence of disorders such as thrombosis, venous thrombosis, and pulmonary embolism. 1 2 3 A main conclusion of these studies is that, although it cannot be proven that the hypercoagulable state is a direct causative effect of SARS-CoV-2 infection, nonetheless it is apparent that patients with SARS-CoV-2 could have a predilection to the occurrence of thromboembolic events. 1

However, currently there are no hypotheses or data that might suggest a molecular mechanism that relates to such SARS-CoV-2-related thromboembolic events. Searching for possible mechanisms, the present study analyzes the SARS-CoV-2 spike glycoprotein (gp) for peptide sharing, that is, molecular mimicry, with human proteins, alterations of which may cause thromboses and hemostasis diseases. The underlying scientific rationale is that peptides common to a pathogen and the human host may lead to autoimmune pathologies through cross-reactivity phenomena following pathogen infection. 4 5 6 The results indicate that several linear sequences shared between the SARS-CoV-2 spike gp and human proteins related to thromboembolic events can possibly generate pathogenic autoantibodies via cross-reactivity and immunologic imprinting phenomena, in this way leading to thromboses and hemostasis disorders.

## Materials and Methods

Peptide sharing between spike gp (NCBI, GenBank Protein Accession, ID: QHD43416.1) from SARS-CoV-2 and human proteins related to thromboses and hemostasis disorders was analyzed as previously detailed. 4 5 6 In brief, pentapeptides were used as sequence probes since a peptide grouping formed by five amino acid (aa) residues defines a minimal immune determinant that can (1) induce highly specific antibodies, and (2) determine antigen–antibody specific interaction. 7 8 Human proteins linked to thromboses and hemostasis disorders were retrieved from UniProtKB database ( *www.uniprot.org* ). 9 Methodologically the spike gp primary sequence was dissected into pentapeptides offset by one residue (i.e., MFVFL, FVFLV, VFLVL, FLVLL, and so forth) and the resulting viral pentapeptides were analyzed for occurrences within the human proteins related to thromboses and hemostasis disorders. Then, the shared peptides were also controlled for occurrences in the pathogens *Bordetella pertussis* , *Corynebacterium diphtheriae, Clostridium tetani* , *Haemophilus influenzae* , and *Neisseria meningitidis* .

The immunological potential of the peptides shared between SARS-CoV-2 spike gp and thrombosis-related proteins was analyzed by searching the Immune Epitope DataBase (IEDB [ *www.iedb.org/* ]) 10 for immunoreactive SARS-CoV-2 spike gp-derived epitopes hosting the shared pentaptides.

## Results and Discussion

### Peptide Sharing between SARS-CoV-2 Spike Glycoprotein and Thromboses-Related Human Proteins

Table 1 shows that 60 minimal immune determinants are shared between SARS-CoV-2 spike gp and 44 human proteins that—when altered, mutated, deficient or, however, improperly functioning—may cause diseases that include coagulation disorders, bruising, bleeding, hemorrhages, retinal vessel occlusion, cerebral thrombosis, venous thrombosis, ischemic stroke, and thrombophilia, inter alia.

**Table 1:** Pentapeptide sharing between SARS-CoV-2 spike gp and human proteins linked when altered, mutated, or deficient to blood diseases

Shared peptides	Human proteins and associated functions/pathologies a b	References
MTKTS, NLLLQ	*ADTRP* ( *androgen-dependent TFPI-regulating protein* ) Regulates the anticoagulant activity of the tissue factor pathway inhibitor, dysfunctions of which lead to vascular diseases	11
TQLPP, PRTFL	*ALG12* : *Dol-P-Man: Man(7)GlcNAc(2)-PP-Dol α-1,6-mannosyltransferase* Psychomotor retardation, hypotonia, coagulation disorders, and immunodeficiency	12
SAIGK	*ALG8* : *Dolichyl pyrophosphate Glc1Man9GlcNAc2 α-1,3-glucosyltransferase* Pathologies: see ALG12 above	13
AEIRA	*ANXA6* ( *annexin A6* ) Anticoagulant protein from human placenta	14
QLIRA, IRASA	*AP3B1* ( *AP-3 complex subunit β-1* ) Associates with Hermansky–Pudlak syndrome. Bleeding diathesis resulting in bruising, epistaxis, gingival bleeding, postpartum hemorrhage, bleeding	15
LIGAE	*APLP2* ( *amyloid-like protein 2* ) The soluble form may have inhibitory properties toward coagulation factors and regulates cerebral thrombosis	16
VLLPL	*B3AT* ( *band 3 anion transport protein* ) Involved in venous thrombosis of unknown origin	17
FGGVS	*B4GT1* ( *β-1,4-galactosyltransferase 1* ) Defects in the nervous system development, psychomotor retardation, dysmorphic features, hypotonia, coagulation disorders	18
KGYHL	*C4BPB* ( *C4b-binding protein β chain* ) Controls complement activation; binds as a cofactor to C3b/C4b inactivator; possibly involved in the susceptibility to venous thrombosis	19 20
LTVLP	*CBS* ( *cystathionine β-synthase* ) CBS-deficient patients are prone to vascular thrombosis	21
NSVAY	*CO1A1* ( *collagen α-1(I) chain* ) Connective tissue disorders characterized by fragile, bruisable skin	22 23
PGQTG, NGLTG	*CO1A2* ( *collagen α-2(I) chain* ) Pathology: see CO1A1 above	22 23
TQSLL, GTGVL	*COG1* ( *conserved oligomeric Golgi's complex subunit 1* ) Psychomotor retardation, hypotonia, coagulation disorders, and immunodeficiency	24
STNLV, GAISS	*COG2.* ( *conserved oligomeric Golgi's complex subunit 2* ) Pathology: as for COG1	25
PINLV	*COG5* ( *conserved oligomeric Golgi's complex subunit 5* ) Pathology: as for COG1	26
LPFQQ, PFQQF, IGKIQ	*ENTP1* ( *ectonucleoside triphosphate diphosphohydrolase 1* ) Implicated in the prevention of platelet aggregation	27 28
YTSAL	*EPHB2* ( *ephrin type-B receptor 2* ) Regulation of platelet activation and blood coagulation	29
VLNDI	*F13A* ( *coagulation factor XIII A chain* ) Relates to hematologic disorders characterized by bleeding tendency	30
DPLQP	*FA5* ( *coagulation factor V* ) Central regulator of hemostasis. Parahemophilia, i.e., poor clotting; pregnancy loss, ischemic stroke, thrombophilia	31 32 33 34
PPLLT, FVTQR	*FA8* ( *coagulation factor VIII* ) Hemophilia	35
NSYEC	*FA9* ( *coagulation factor IX* ) Hemophilia	35
SSANN	*FIBA* ( *fibrinogen α chain* ) Bleeding, amyloidosis, arterial hypertension, hepatosplenomegaly, cholestasis, petechial skin rash; thromboembolic complications	36 37 38
GAGAA	*GATA4* ( *transcription factor GATA-4* ) Regulates factor X, a vitamin K-dependent serine protease that functions in blood coagulation. Can predispose to dilated cardiomyopathy, and to premature death	39 40 41
NDPFL	*GP1BA* ( *platelet glycoprotein Ib α chain* ) Epistaxis; hemorrhage; menorrhagia; purpura; congenital bleeding diathesis; large platelets; thrombocytopenia; long bleeding time	42
ALLAG	*GPIX* ( *platelet glycoprotein IX* ) Epistaxis; hemorrhage; menorrhagia; purpura; congenital bleeding diathesis; large platelets; thrombocytopenia; long bleeding time	42
KLIAN	*HABP2* ( *hyaluronan-binding protein 2* ) Serine protease involved in coagulation fibrinolysis and inflammatory pathways	43
TQLPP	*HPS4* ( *Hermansky–Pudlak syndrome 4 protein* ) Epistaxis; reduced visual acuity; horizontal nystagmus; iris transillumination; restrictive lung disease; bruising; bleeding tendency; menorrhagia; absence of platelet dense bodies; lack of secondary aggregation response of platelets	44
HTSPD	*HPS5* ( *Hermansky–Pudlak syndrome 5 protein* ) As HPS4 above	45
FNATR, DRLIT	*HS3S5* ( *heparan sulfate glucosamine 3-O-sulfotransferase 5* ) Catalyzes a crucial step in the biosynthesis of the anticoagulant heparan sulfate	46
SASFS	*ITA2* ( *integrin α-2* ) Associates with increased ischemic stroke risk; thrombophilia	47 48
VRDLP	*ITB3* ( *integrin β-3* ) Thrombasthenia, characterized by mucocutaneous bleeding	49
FGTTL, YDPLQ, GDISG	*JAK2* ( *tyrosine-protein kinase JAK2* ) Thrombophilia, thrombocytosis	50 51
VNLTT, GDSSS, VTYVP	*MMRN1* ( *multimerin-1* ) Deficiency in multimerin-1 associates with bleeding disorder	52
LLPLV	*PLF4 (Platelet factor 4)* Involved in thrombosis	53
TFGAG	*PLMN* ( *plasminogen* ) may be associated with susceptibility to thrombosis	54
TVEKG, TGTGV	*PROS* : *vitamin K-dependent protein S* Anticoagulant plasma protein. Helps to prevent coagulation and stimulates fibrinolysis. Deficiency leads to impaired blood coagulation and a tendency to venous thrombosis	55 56
LALHR	*PROZ* : *vitamin K-dependent protein Z* Helps hemostasis by binding thrombin and promoting its association with phospholipid vesicles. Deficiency may be a risk factor for retinal vessel occlusion	57
IDRLI	*PTPRJ* : *receptor-type tyrosine-protein phosphatase η* Lack of PTPRJ leads to a bleeding tendency and defective arterial thrombosis	58
VFAQV	*TF* ( *tissue factor* ): Initiates blood coagulation by forming a complex with circulating factor VII or VIIa	59
LFRKS	*THRB* : *Prothrombin* : Functions in blood homeostasis	60
AGAAL, GAALQ	*TRBM* ( *thrombomodulin* ) Relates to thrombophilia, venous thrombosis, and thromboembolic disease. TRBM administration is beneficial in sepsis-induced coagulopathy and in disseminated intravascular coagulations	61 62 63
TLLAL	*TSP1* ( *thrombospondin-1* ): Coronary artery disease	52 64
TLLAL, SCGSC	*TSP2* ( *thrombospondin-2* ): Coronary artery disease	52 64
VSSQC, LQYGS	*VWF (von Willebrand factor)* Von Willebrand's disease is characterized by deficiency of circulating VWF that is otherwise structurally and functionally normal. Clinical features: impaired platelet aggregation, cardiovascular diseases, mucocutaneous bleeding, epistaxis, menorrhagia	52 65 66 67

Abbreviations: gp, glycoprotein; SARS-CoV-2, severe acute respiratory syndrome-coronavirus-2.

a Human proteins given by Uniprot accession and name in italics.

b Functions and/or pathologies: data and further references from Uniprot, PubMed, and OMIM databases

### Immunological Potential of the Viral versus Human Peptide Sharing

The data shown in Table 1 are quantitatively impressive and become strikingly preeminent from a pathological perspective when analyzed for their immunological potential. Indeed, exploration of the IEDB 10 reveals that nearly all the shared pentapeptides described in Table 1 are also disseminated among SARS-CoV-2 spike gp-derived epitopes that have been experimentally validated as immunoreactive and are cataloged at the IEDB database ( *http://www.iedb.org* ). 10

That is, Table 2 concretely supports the possibility that autoimmune cross-reactions may be triggered by SARS-CoV-2 infection/active immunization and hit human proteins related to thrombotic/thromboembolic disorders and coagulopathies, inter alia. Clinically, the vastity of the potential immunological cross-reactivity that emerges from Table 2 indicates that mild-to-moderate and severe forms of thrombosis and coagulopathy may unavoidably accompany SARS-CoV-2 infection/active immunization.

**Table 2:** Distribution of peptides shared between SARS-CoV-2 spike gp and human proteins related to thromboses and hemostasis disorders among 94 experimentally validated SARS-CoV-2 spike gp-derived epitopes

ID a	Epitope b	ID a	Epitope b
1069137	aqYTSALLAGtitsg	1309555	qcVNLTTrTQLPPaytnsft
1069290	ctlksfTVEKGiyqt	1309558	qfnSAIGKIQdslsstasal
1071585	nlVRDLPqgfsalep	1309564	qtragcLIGAEhvnNSYECd
1071723	patvcgpkkSTNLVknkc	1309573	rLFRKSnlkpferdisteiy
1072807	skhtPINLVRDLPqg	1309595	tnftisvtteilpvsMTKTS
1072965	svtteilpvsMTKTS	1309598	tvYDPLQPeldsfkeeldky
1073281	tesnkkfLPFQQFgrdia	1309599	Tyvpaqeknfttapaichdg
1073938	vqIDRLITgrlqslq	1309600	tyvtqQLIRAAEIRASAnla
1074201	ylyrLFRKSnlkpfe	1309602	vcgpkkSTNLVknkcvnfnf
1074838	AEIRASAnlaatk	1309603	vknkcvnfnfNGLTGTGVLt
1074925	hVTYVPaqeknf	1309604	VLNDIlsrldkveaevqidr
1074969	lgaeNSVAYsnn	1309621	yskhtPINLVRDLPqgfsal
1074974	lLALHRsyl	1310254	aeNSVAYsnnsiaip
1075005	nqKLIANqf	1310281	aphgvvflhVTYVPa
1075031	rLFRKSnlk	1310303	caqkfngLTVLPpll
1075039	rqiaPGQTGkiadynykl	1310336	dskTQSLLivnnatn
1075066	sVLNDIlsrl	1310392	FGTTLdskTQSLLiv
1075079	tPINLVrdl	1310401	fkiyskhtPINLVrd
1075085	tvYDPLQPeldsfk	1310415	fngLTVLPPLLTdem
1075094	vlPPLLTdemiaqyt	1310434	GAISSVLNDIlsrld
1075125	ysvlynSASFStfk	1310444	givnntvYDPLQPel
1075131	yyvgylqPRTFLl	1310487	iginitrfqTLLALh
1087680	PINLVRDLPqgfsalepl	1310506	irgwiFGTTLdsktq
1125063	gLTVLPpll	1310513	itrfqTLLALHRsyl
1309117	ggnynylyrLFRKSn	1310592	lLALHRsyltpgdss
1309118	gpkkSTNLVknkcvn	1310611	lPPLLTdemiaqyts
1309123	khtPINLVRDLPqgf	1310633	lyenqKLIANqfnsa
1309140	tdemiaqYTSALLAG	1310787	SASFStfkcygvspt
1309147	ylqPRTFLl	1310828	svlynSASFStfkcy
1309418	AEIRASAnlaatkmsecvlg	1310852	tlvkqlssnfGAISS
1309442	ayyvgylqPRTFLlkyneng	1310865	trfqTLLALHRsylt
1309450	dplsetkctlksfTVEKGiy	1310899	VLLPLVSSQCVNLTT
1309451	dsfkeeldkyfknHTSPDvd	1310909	VNLTTrTQLPPaytn
1309461	ehvnNSYECdipigagicas	1310927	vtqnvlyenqKLIAN
1309464	esnkkfLPFQQFgrdiadtt	1310947	wTFGAGAALQipfam
1309469	fknHTSPDvdlGDISGinas	1310979	yvgylqPRTFLlkyn
1309470	fknidgyfkiyskhtPINLV	1311657	ccSCGSCckfdeddsepvlkgvkl
1309475	gccSCGSCckfdeddsepvl	1311813	rLFRKSnlkp
1309492	ilditpcsFGGVSvitpgtn	1313244	nSASFStfk
1309506	kvggnynylyrLFRKSnlkp	1313285	PINLVRDLPqgfsal
1309515	lhrsyltpGDSSSgwtagaa	1313286	PINLVRDLPqgfwal
1309516	litgrlqslqtyvtqQLIRA	1314023	ynylyrLFRKSnlkp
1309523	lssnfGAISSVLNDIlsrld	1317916	gylqPRTFLl
1309524	lyenqKLIANqfnSAIGKIQ	1321084	lPPLLTdem
1309531	NGLTGTGVLtesnkkflpfq	1327418	vYDPLQPeldsf
1309532	ngLTVLPPLLTdemiaqyts	1327923	yenqKLIANqf
1309534	nitrfqTLLALHRsyltpgd	1328800	ytmslgaeNSVAY

Abbreviations: gp, glycoprotein; SARS-CoV-2, severe acute respiratory syndrome-coronavirus-2.

a Epitopes listed as the Immune Epitope DataBase ID.

b Shared peptides given in capital letters.

### Autoimmunity Potential and the Immunological Memory

As already highlighted also in other infection models, 68 69 70 71 one has to consider that immunologic memory can powerfully enhance and amplify the autoimmune cross-reactivity potential because of interpathogen peptide sharing. Indeed, as a rule, the immune system recalls preexisting memory responses toward past infections rather than inducing ex novo responses toward the recent ones since hallmark of the immune system is the memory for the immune determinants it has previously encountered. 72 73

Here, comparative sequence analyses show that 31 out of the 60 minimal immune determinants common to SARS-CoV-2 spike gp and human proteins related to thromboses are also widespread in pathogens, such as *B. pertussis* , *C. diphtheriae, C. tetani* , *H. influenzae* , and *N. meningitidis* , that are in pathogens with which, in general, an individual has already come into contact during his life due to infections or by vaccination ( Table 3 ).

**Table 3:** Occurrence in microbial organisms of pentapeptides common to SARS-CoV-2 spike gp, SARS-CoV-2 spike gp-derived epitome, and human proteins related to thromboses, coagulopathies, and hemostasis disorders

Organism	Shared peptides
*Bordetella pertussis*	AEIRA, AGAAL, ALLAG, GAALQ, GAGAA, LLPLV, PFQQF, QLIRA, SSANN, TGTGV, VLLPL, YDPLQ
*Corynebacterium diphtheriae*	AEIRA, AGAAL, ALLAG, DPLQP, GAALQ, GAGAA, GTGVL, LLPLV, TVEKG
*Clostridium tetani*	AGAAL, LQYGS
*Haemophilus influenzae*	AEIRA, AGAAL, FGGVS, GAALQ, GAGAA, GTGVL, KLIAN, LALHR, LLPLV, LPFQQ, LTVLP, NLLLQ, NSVAY, TLLAL, TQSLL, VLLPL, VLNDI, VNLTT, YTSAL
*Neisseria meningitidis*	AEIRA, AGAAL, ALLAG, DRLIT, GAALQ, GAGAA, IDRLI, KLIAN, LALHR, LTVLP, PINLV, TLLAL, VLLPL, VLNDI

Abbreviations: gp, glycoprotein; SARS-CoV-2, severe acute respiratory syndrome-coronavirus-2.

Hence, Table 3 indicates the possibility that a preexisting immune response to previously encountered pathogens (in the present case: *B. pertussis* , *C. tetani* , *C. diphtheriae, H. influenzae* , and/or *N. meningitidis* ) might be magnified and intensified following SARS-CoV-2 infection/active immunization. That is, immunological imprinting can start a chain of events according to which followings can be measured:

- Following exposure to SARS-CoV-2, the primary response to the virus can turn into a secondary response to previously encountered pathogens of which the immune system has stored an immunological memory.
- The anamnestic secondary and, by definition, extremely powerful response against immune determinants previously encountered implies not only that a low or no immune response will be evoked against the pathogen lastly encountered, that is, SARS-CoV-2, but also entails that the anamnestic secondary reaction against the early sensitizing pathogens—in the case in point, *B. pertussis* , *C. tetani* , *C. diphtheriae* , and/or *N. meningitidis* —will fail because those early sensitizing pathogens are no more present in the organism.
- As a final result, the anamnestic, high affinity, high avidity, and extremely powerful secondary immune response triggered by the lastly encountered pathogen (SARS-CoV-2) and addressed toward past infections may find an outlet by hitting available human targets, that is, in the case in object, the human proteins related to thromboses and hemostasis diseases ( Table 1 ).

## Conclusion

The last decades witnessed the emerging of infectious diseases and, consequently, intensive application of immunization procedures. Concomitantly, concerns about possible adverse events have increased. A recent crucial example is the immunization campaign with the dengue vaccine that highlighted the risk of enhanced disease after vaccination. 74

Today, the clinical context associated with SARS-CoV-2 infection/active immunization is no different. Actually, understanding whether undesired collateral events, such as the thrombotic manifestations and bleeding disorders discussed in this study, may causally associate with the viral infection/active immunization is a fundamental step for fighting the current pandemic. In this context, the present study:

- Analyzed the hypothesis that infectious agents can induce cross-reactive autoantibodies capable of hitting and altering human proteins that regulate hemostasis and coagulation.
- Showed that numerous peptides endowed with an immunologic potential are common to SARS-CoV-2 spike gp and human proteins, when mutated, altered, deficient or improperly functioning, are associated with thromboses and hemostasis diseases ( Tables 1 and 2 ).
- Documented that the peptide commonality extends to pathogens that usually have been already encountered by an individual during his life ( Table 3 ).

Scientifically, the data indicate that peptide sharing–associated cross-reactivity and, in conjunction, immunological imprint might help explain some of the thromboembolic events that rapidly, massively, and violently may arise following SARS-CoV-2 infection/active immunization.

Clinically, the present data warrant testing of patients' sera for autoantibodies against the peptide targets described in Tables 1 2 and 3 , and reiterate the suggestion advanced already in 2000 75 that immunotherapies should take advantage of the principle of peptide uniqueness, that is, of peptides present in the antigen of interest and absent in the human proteome. 71 76 77 78 79 80 81
